# Development of an Acute Method to Deliver Transgenes Into the Brains of Adult *Xenopus laevis*

**DOI:** 10.3389/fncir.2018.00092

**Published:** 2018-10-26

**Authors:** Ayako Yamaguchi, Diana J. Woller, Paulo Rodrigues

**Affiliations:** Department of Biology, University of Utah, Salt Lake City, UT, United States

**Keywords:** viral vector, vesicular stomatitis virus, *Xenopus laevis*, transgene, neurons, vocalizations, central pattern generator, electroporation

## Abstract

The central vocal pathway of the African clawed frog, *Xenopus laevis*, is a powerful vertebrate model to understand mechanisms underlying central pattern generation. However, fast and efficient methods of introducing exogenous genes into the neurons of adult *X. laevis* are currently not available. Here, we systematically tested methods of transgene delivery into adult *X. laevis* neurons. Although successfully used for tadpole neurons for over a decade, electroporation was not efficient in transfecting adult neurons. Similarly, adeno-associated virus (AAV) was not reliable, and lentivirus (LV) failed to function as viral vector in adult *Xenopus* neurons. In contrast, vesicular stomatitis virus (VSV) was a fast and robust vector for adult *X. laevis* neurons. Although toxic to the host cells, VSV appears to be less virulent to frog neurons than they are to mice neurons. At a single cell level, infected neurons showed normal physiological properties up to 7 days post infection and vocal circuits that included infected neurons generated normal fictive vocalizations up to 9 days post infection. The relatively long time window during which the physiology of VSV-infected neurons can be studied presents an ideal condition for the use of optogenetic tools. We showed that VSV does not gain entry into myelinated axons, but is taken up by both the soma and axon terminal; this is an attractive feature that drives transgene expression in projection neurons. Previous studies showed that VSVs can spread across synapses in anterograde or retrograde directions depending on the types of glycoprotein that are encoded. However, rVSV did not spread across synapses in the *Xenopus* central nervous system. The successful use of VSV as a transgene vector in amphibian brains not only allows us to exploit the full potential of the genetic tools to answer questions central to understanding central pattern generation, but also opens the door to other research programs that focus on non-genetic model organisms to address unique questions.

## Introduction

Understanding the neural mechanisms underlying behavior presents a difficult challenge requiring a well-chosen model system and sophisticated experimental tools. Vocalizations of the African clawed frog, *Xenopus laevis* are an exceptionally well-suited model system for this objective for the following reasons. First, a simplified mechanism of vocal production allows straightforward interpretations of neuronal activity with respect to behavior (Yamaguchi and Kelley, 2000). Second, neural mechanisms of calling can be studied *in vitro* because fictive vocalizations can be elicited in the isolated brain of adults (Rhodes et al., 2007), an example only found in select few vertebrate species. Third, the vocalizations of female *X. laevis* can be rapidly masculinized in an androgen-dependent manner (Potter et al., 2005), providing us with a unique opportunity to explore neural plasticity. Despite these unique advantages, genetic tools that have revolutionized the field of neuroscience in recent years have largely not been available to the central nervous system (CNS) of adult *X. laevis.* Genetically encoded tools, including optogenetic sensors and actuators, offer exciting possibilities to characterize and manipulate the activity of a select population of neurons. Creating transgenic organisms is labor-intensive, expensive and time-consuming, especially in *X. laevis* due to its long generation time. In parallel to an ongoing effort to create transgenic *X. laevis* lines at the National Xenopus Resource and elsewhere in the world, the development of acute transfection-mediated gene expression methods is desirable. Here, we explored techniques to express exogenous genes in adult neurons of *X. laevis*.

Electroporation has been successfully used to deliver exogenous genes into the brains of a variety of species *in vivo* (Wang and Mei, 2013; Ahmadiantehrani and London, 2017), including *X. laevis* tadpoles (Haas et al., 2001, 2002). Recently, a method has been described to introduce exogenous genes into a restricted region of the adult brains of zebrafish, greatly enhancing the utility of the technique (Zou et al., 2014). In this study, we examined the utility of the electroporation technique in adult *X. laevis*.

Direct injection of viral vectors into the brains allows rapid expression of exogenous genes in a spatially defined area. Although viral gene transfer is a well-established method in mammals, it is rarely used in lower vertebrates, with the exception of zebrafish. Previously, a handful of attempts have been made to use viral vector to deliver exogenous genes into non-dividing neurons of *X. laevis* tadpoles *in vivo*. These viruses include recombinant vaccinia virus (Wu et al., 1995) and adenovirus (Dutton et al., 2009). Unfortunately, whether these viruses transduce adult neurons has not been tested to date. Here, we systematically tested the efficiency of three neurotropic viral vectors that infect non-dividing cells; adeno-associated virus (AAV), lentivirus (LV), and vesicular stomatitis virus (VSV). AAV and LV are by far the most commonly used viral vectors in neuroscience. They are particularly attractive because a large repertoire of recombinant viruses is currently available. AAV is a small single-stranded, non-pathogenic DNA virus that is reported not to be host-specific when injected directly into the brain (Janson et al., 2001). LV is a single-strand RNA virus whose native host range only includes mammals. However, the host range of LV can be greatly extended by pseudotyping LV with glycoprotein of VSV. Finally, we used VSV, a single-strand negative RNA virus with a broad tropism that has been proven to transduce neurons of a large variety of species including mammals, fish, and invertebrates (van den Pol et al., 2009; Mundell et al., 2015). VSV uptake mostly takes place at cell bodies and the proximal dendrites of the host neuron (i.e., direct infection). Accordingly, reporter gene proteins are first detected in cell bodies, and spread across axons at a later time (van den Pol et al., 2009). Unlike AAV and LV, VSV viral mRNA and protein synthesis occurs within the cytoplasm of the host cell without the transport of viral genome to the host nucleus (Heinrich et al., 2010). Therefore, infected cells release the first progeny virus in as little as 1 h (van den Pol et al., 2009). Although VSV is known to be lethal to the host cells, electrophysiological recordings can be obtained from infected neurons expressing exogenous genes within a day of infection in mice (van den Pol et al., 2009; Beier et al., 2013), indicating that there is a window of time during which the infected neuron with exogenous gene product remain healthy. Whether the virulence of VSV is the same for different host species has never been systematically tested to date. VSVs have been reported to spread transsynaptically to pre- or post-synaptic neurons depending on the types of glycoprotein encoded (Beier et al., 2011, 2013, 2016). In these reports, rVSV encoding rabies glycoprotein spread across synapses retrogradely, whereas those encoding the glycoprotein of VSV spread transsynaptically in the anterograde direction. We tested whether the VSV also spread transsynaptically among neurons of adult *X. laevis*.

In the present study, we found that electroporation does not lead to efficient exogenous gene expression in neurons of adult *X. laevis*. Similarly, AAV was not a reliable viral vector, and LV failed to work as a viral vector in adult neurons of *X. laevis*. In contrast, VSV proved to be a quick and effective vector to deliver transgenes in the neurons of adult *X. laevis*. We explored the physiological function of the VSV infected neurons, the locus of viral uptake by the neurons, and the possibility of viral spread across synapses in adult *X. laevis*. Given the broad host range of VSV, fast and robust expression of transgenes, and prolonged health of infected neurons, we expect that this vector is a powerful means to make genetically encoded tools accessible to a variety of non-genetically model species.

## Materials and Methods

A summary of all the experiments is shown in Table 1.

**Table 1:** Summary of animals used for the experiments.

Procedure	Virus/plasmid type	Animals	Injection location	Sample size	Time after procedure (sample size)	Time unit
Electroporation, *in vivo*	pPB.CAG.GFP	Tadpoles	OB/T/OT	11	16	h
	pCS2.EGFP(CAAX)	Tadpoles	OB/T/OT	7	16	h
	pCS2FA.ChR2.YFP	Tadpoles	OB/T/OT	13	24	h
	pCS2FA.ChR2.YFP	Frogs	OB/T/OT	8	5 (2), 6 (6)	h
Electroporation, *in vitro*	pPB.CAG.GFP	Frogs	OB/T/OT	1	27	h
	pCS2.EGFP(CAAX)	Frogs	OB/T/OT	5	16 (3), 22 (2)	h
	pCS2FA.ChR2.YFP	Frogs	OB/T/OT	5	2 (1), 3 (3), 4 (1)	d
AAV injection, *in vivo*	AAV9.hSyn.hChR2(H134R)eYFP.WPRE.hGH	Frogs	OB/T/OT	34	21	d
	retroAAV mCherry-Cre	Frogs	OB/T/OT	8	21	d
	AAV2/1.Syn.ChR2(H134R)eYFP.AWP.hGH	Frogs	OB/T/OT	3	21	d
	AAV-PHPeB:Cag-mNeonGreen	Frogs	OB/T/OT	4	21	d
LV injection, *in vivo*	LV-SIN-CMV-eGFP	Frogs	OB/T/OT	4	6	w
	EIAV-TLoop-ChR2-YFP	Frogs	OB/T/OT	9	12	w
	EIAV-TLoop-ChR2-YFP	Tadpoles	OB/T/OT	9	12	d
VSV injection, *in vivo*	VSV(VSV-G) plaque 14	Frogs	OB/T/OT	8	2 (4), 4 (2), 6 (2)	d
	VSV(VSV-G) plaque 14	Frogs	n.IX-X	6	2 (2), 5 (1), 6 (1), 7 (2)	d
	VSV(VSV-G) plaque 21	Frogs	OB/T/OT	28	2 (10), 4 (3), 7 (4), 10 (11)	d
	VSV(VSV-G) plaque 21	Frogs	n.IX-X	18	3	d
	VSV(VSV-G) plaque 21	Frogs	nerve IX-X	7	2 (1), 28 (4), 35 (2)	d
	VSV(VSV-G) plaque 21	Tadpoles	OB/T/OT	42	1 (9), 2 (26), 4 (4), 5 (3)	d
	VSV(VSV-G) plaque 21	Tadpoles	nose	85	1 (2), 2 (29), 3 (1), 4 (22), 5 (31)	d
	VSV(RABV-G)	Frogs	OB/T/OT	7	1 (1), 2 (4), 3 (2)	d
	VSV(RABV-G)	Frogs	n.IX-X	8	2 (3), 8 (1), 9 (2), 10 (2)	d
	VSV(RABV-G)	Frogs	laryngeal muscles	10	21 (1), 22 (1), 23 (4), 27 (2), 28 (2)	d
	VSV(RABV-G)	Frogs	gastrocnemius muscles	4	7	d
	VSV(RABV-G)	Tadpoles	OB/T/OT	12	3	d
	G-Deleted VSV-tdTomato	Frogs	OB/T/OT	3	2	d
	G-Deleted VSV-tdTomato	Frogs	n.IX-X	1	2	d
	G-Deleted VSV-ChR2YFP	Frogs	OB/T/OT	7	2 (4), 5 (3)	d
	G-Deleted VSV-ChR2YFP	Frogs	n.IX-X	2	9 (1), 10 (1)	d

*Injection site indicates the location where plasmid or virus was injected. “Time after procedure” column indicates the interval between the plasmid electroporation/viral injection and the time when brain was fixed, indicated in hours (h), days (d), or weeks (w) in the “time unit” column. When animals in an experimental group had variable amount of survival time, the number of animals sacrificed at each time point is shown in parenthesis in “Time after procedure” column. Injection location within the brain was separated into region rich in cell bodies including olfactory bulb, telencephalon, and optic tectum (OB/T/OT) and the region rich in myelinated axons, the laryngeal motor nucleus in the brainstem (n.IX-X). AAV, adeno-associated virus; LV, lentivirus, OB/T/OT, olfactory bulb/telencephalon/optic tectum; n.IX-X, laryngeal motor nucleus; VSV, vesicular stomatitis virus.*

### Animals

One hundred and ninety adult *Xenopus laevis* (average weight and length, 27.14 g, 6.54 cm) and 179 tadpoles were obtained from Nasco (Fort Atkinson, WI, United States). Tadpoles were kept in 0.1X Steinberg solution, and animals between stages 46 and 47 were used for the experiments. Adult frogs were kept in reverse osmosis water conditioned for chlorine, chloramine, and ammonia, added with aquarium salt. All animals were kept in 12 h photoperiod at 22°C. The stock density of frogs was 2 L per frog, and that of tadpoles was 150 mL per tadpole. The length of time the animals remained in captivity were 4 to 5 days for tadpoles and from 1 week to 4 months for adult frogs. All the procedures were approved by the Institutional Animal Care and Use Committee at the University of Utah.

### Electroporation of Plasmids

**FIGURE 1:**
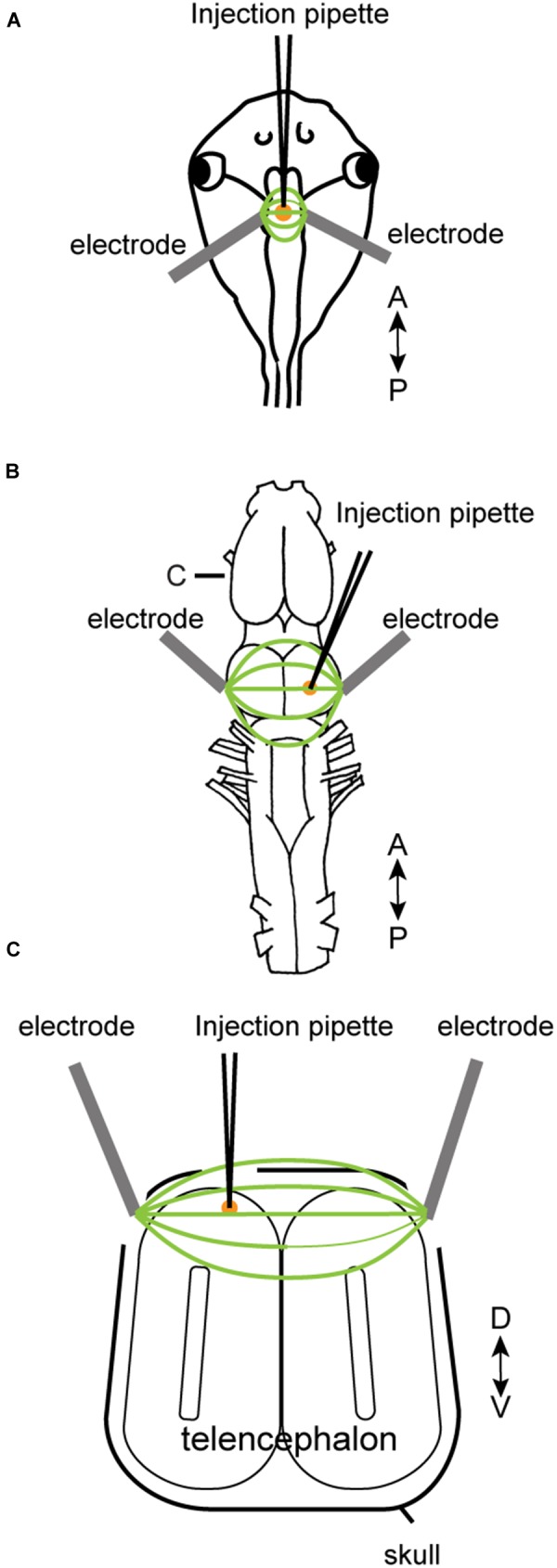
Electroporation setup used for tadpoles *in vivo* **(A)**, for adult brain *in vitro* **(B)**, and adult brain *in vivo* **(C)**. A glass pipette containing plasmid suspension was lowered into the brain **(A–C)**, and plasmid was pressure-injected. Following the injection, electrical pulses were applied to a pair of platinum electrodes placed on the two sides of the injection area. A small amount of calcium-free Steinberg solution (tadpoles) or frog saline (adults) were applied to cover the tips of the two electrodes along with the injection area. **(C)** Transverse section of the telencephalon at the level indicated as **(C)** in **(B)**.

To electroporate plasmids into tadpole brains, animals were first placed in 0.02% tricaine methanesulfonate (MS-222, Sigma) in 0.1X Steinberg solution. When the animal was deeply anesthetized, it was placed in a dish and a drop of calcium-free 0.1X Steinberg solution was applied to the head. The plasmid suspension (Table 2) was injected into the ventricle of tadpoles via glass pipette, and a pair of platinum electrodes (1 mm apart) were lowered to sandwich the ventricle from both lateral sides (Figure 1A). Immediately after the injection, electrical pulses (70 ms duration, 20 V) were delivered five times at 1 Hz.

**Table 2:** Types of plasmids used for electroporation.

Plasmid name	Promoter	Transgene	Concentration (μg/μL)
pPB.CAG.GFP	CAG	GFP	3.5
pCS2.EGFP(CAAX)	sCMV	EGFP	1.1
pCS2FA.ChR2.YFP	sCMV	ChR2YFP	1.7

*sCMV, simian cytomegalovirus promoter; GFP, green fluorescent protein; EGFP, enhanced green fluorescent protein.*

Electroporation of adult brains were carried out both *in vitro* and *in vivo*. Animals were first deeply anesthetized by injecting 1 mL of 1.3% MS-222 subcutaneously. For *in vitro* electroporation, the brains were isolated and pinned to a petri-dish coated with silicone elastomer (Sylgard; Dow Corning) filled with calcium-free saline solution. Plasmid suspension (500 nL) was pressure-injected into the olfactory bulb, telencephalon, or optic tectum using a glass pipette. Immediately after the injection, electrical current pulses were applied through a pair of tungsten or platinum electrodes (3.5 mm apart) that surround the injection site (Figure 1B). Current pulses used were between 70 and 100 V, 25 to 70 ms in duration, and was repeated 10 to 12 times (both directions of electric field were applied by reversing polarity after 5 or 6 pulses) at 1 Hz. To compensate for the longer distance between the two electrodes accommodating the larger size of the adult brains (3.5 mm between the two electrodes for adults as opposed to 1 mm for tadpoles), we increased the voltage to at least 70 V to maintain constant electric field strength to a minimum of 20 V/cm. For *in vivo* electroporation, a small round craniotomy was made over the dorsal telencephalon of a deeply anesthetized animal using a dental drill with a bur diameter of 0.5 mm, and a thin longitudinal craniotomy was made on both side of the initial round craniotomy to insert platinum electrodes (Figure 1C). A glass pipette (the tip diameter of 10 to 20 μm) containing plasmid suspension was lowered into the round craniotomy. Immediately after the plasmid injection (typically within 30 s), 14 current pulses (70 ms, 70 V positive and negative polarity, seven times each) at 1 Hz was applied between a pair of platinum electrodes placed on both side of the craniotomy.

### Viral Injection

The viral vectors used in this study are listed in Table 3. AAV-PHPeB:Cag-mNeonGreen was kindly made available to us by Dr. Viviana Gradinaru, CalTech, and all the other AAV vectors were obtained from the viral vector cores of the University of Pennsylvania. The remaining viral vectors (LV, EIAV, VSV) were obtained from the GT3 core of the Salk Institute. To inject virus directly into the brains, a craniotomy was made onto the dorsal surface of the skull of a deeply anesthetized animal using a dental drill (bur diameter 0.5 mm). A glass pipette containing viral suspension with a tip diameter between 10 and 20 μm was lowered into the brain using stereotaxic manipulator (David Kopf Instruments), and 300–700 nL of viral suspension was pressure-injected into the brain at a rate of ∼100 nL per minute using Picospritzer III (Parker Hannifin). Pressure used for the injection ranged from 20 to 25 psi and the pressure pulse duration ranged from 3 to 15 ms. After the injection was completed, the pipette was left in the same target position for 10 min and was retracted slowly thereafter. For all the procedures described above involving DNA and viruses, standard institutional safety procedures approved by the University of Utah have been followed.

**Table 3:** Types of viruses used as a vector to deliver transgenes.

Virus type	Recombinant virus name	Promoter	Transgene	Titer (TU/mL)
AAV	AAV9.hSyn.hChR2(H134R)eYFP.WPRE.hGH	hSynapsin	hChR2-YFP	2.75E^+13^
AAV	retroAAV mCherry-Cre	hSynapsin	mCherry	3.90E^+12^
AAV	AAV2/1.Syn.ChR2(H134R)eYFP.AWP.hGH	hSynapsin	YFP	1.02E^+12^
AAV	AAV-PHPeB:Cag-mNeonGreen	CAG	mNeonGreen	3.46E^+12∗^
LV	LV-SIN-CMV-eGFP	CMV	eGFP	3.20E^+10^
LV(EIAV)	EIAV-TLoop-ChR2-YFP	CMV	ChR2-YFP	2.20E^+10^
VSV	VSV-G VSV-Venus 1 (plaque 14)	n.a.	Venus 1	4.60E^+8^
VSV	VSV-G VSV-Venus 2 (plaque 21)	n.a.	Venus 2	7.56E^+10^
VSV	RABV-G VSV-eGFP	n.a.	eGFP	2.51E^+09^
VSV	G-Deleted VSV-tdTomato	n.a.	tdTomato	5.34E^+10^
VSV	G-Deleted VSV-ChR2YFP	n.a.	mCherry, ChR2YFP	5.00E^+8∗^

*AAV, adeno-associated virus; CMV, cytomegalovirus; eGFP, enhanced green fluorescent protein; EIAV, equine infectious anemia virus; hChR2-YFP, human channelrhodopsin2-yellow fluorescent protein; hSynapsin, human synapsin; LV, lentivirus; VSV, vesicular stomatitis virus; YFP, yellow fluorescent protein. ^∗^gc/mL.*

### Histological Analyses

For histological analyses of tadpole brains, deeply anesthetized animals were placed in 4% paraformaldehyde (PFA) over night at 4°C, and vibratome sectioned into 40 μm thickness (Vibratome series 1000). Similarly, the brains of deeply anesthetized adult frogs were isolated and fixed in 4% PFA for 48 h at 4°C. Fixed adult brains were vibratome sectioned into 80 μm thickness. Gastrocnemius muscles of adult animals injected with rVSV(RABV-G) were isolated from deeply anesthetized animals, fixed in 4% PFA for 48 h at 4°C, placed in 30% sucrose solution overnight, embedded in O.C.T compound (Tissue-Tek), frozen at -80°C, then sectioned into 50 μm thickness using a cryostat (Leica CM1950). We defined fluorescently positive cells to have fluorescent intensity over seven standard deviations above the background fluorescence measured at a location > 1 mm away from the injection site. Labeled neurons in the tissue sections were photographed under an Olympus BX41 microscope with a digital camera (Retiga 2000R, QImaging), a confocal microscope (LSM 880, Zeiss), or a two-photon microscope (Neurolabware), and analyzed using a software (Image-Pro Premier, Media Cybernetics). To compare the efficiency of infection by different virus, we were not able to count the number of labeled neurons because of the variability in the titer of each viral suspension and the volume of viral suspension injected into each animal. Instead, we used the fraction of animals that showed reporter gene expression in the injection site as a measure for infection efficiency.

#### Immunohistochemistry

To identify radial glial cells in adult *X. laevis* brains, we carried out immunohistochemistry for vimentin. Two adult males (6.0, 7.5 cm; 19.9, 35.48 g, respectively) and eight tadpoles (stage 46) were used for this experiment. Brains of deeply anesthetized adult frogs were isolated and fixed in 4% PFA overnight, and cryosectioned in the coronal plane at 35 μm on a cryostat, as described above. Brains of tadpoles were cryosectioned at 20 μm thickness. Tissue sections were mounted directly onto gelatin-coated (i.e., subbed) slides and were then processed for immunohistochemistry. Briefly, sections were incubated in 0.3% Triton-X/phosphate buffer saline (PBS), followed by incubation in a blocking buffer composed of 0.03% Triton-X/PBS and 5% normal goat serum (Thermo Fisher). The primary antibody used was *Xenopus* vimentin antibody (14h7, Developmental Studies Hybridoma Bank, Iowa City, IA, United States), a monoclonal antibody generated in mice immunized with cell residues derived from *Xenopus* kidney epithelial cell line A_6_ that contain vimentin (Dent et al., 1989). Tissue was incubated in the primary antibody at a concentration of 1:50 in 0.3% Triton-X/PBS for 2 h at room temperature, washed in 0.3% Triton-X/PBS and then incubated in AlexaFluor 555 goat anti-mouse secondary antibody (1:200) for 1 h. As a control, adjacent sections from the same brains were processed using all immunohistochemical procedures except for the primary antibody. No staining was observed in the negative control sections. Robust labeling of vimentin-positive radial glial cells in tadpole brains was used as a positive control to verify the validity of the primary antibody and the IHC procedure.

### Electrophysiology

#### Whole-Cell Patch-Clamp Recording From Brain Slice Preparation

Animals (*n* = 6) were anesthetized with injection of MS-222, the brains were isolated, and a 300 μm slice preparation was prepared using a vibratome (Vibratome series 1000) in ice-cold partially frozen saline solution (in mM: 96 NaCl, 20 NaHCO3, 2 CaCl2, 2 KCl, 0.5 MgCl_2_, 10 HEPES, and 11 glucose, pH 7.8). Whole-cell patch-clamp recordings were obtained from reporter gene-positive and -negative neurons using the methods described previously (Yamaguchi et al., 2003). Briefly, brain slices in a recording chamber mounted on a Zeiss Axioskop microscope fitted with infrared-differential interference contrast (IR-DIC) and fluorescent optics were used to visualize labeled and non-labeled neurons while the slices were continuously perfused with oxygenated saline at room temperature (22°C). Labeled and non-labeled neurons were first identified using fluorescence optics, then whole-cell patch-clamp recordings were obtained using IR-DIC optics (Dage MTI CCD 100). Whole-cell patch-clamp pipettes were made from thick-walled (1.5 mm outer diameter; 0.86 mm inner diameter) borosilicate capillaries with a 3 mm trough filament (P-97 microelectrode puller, Sutter). The pipette resistance ranged between 4 and 10 MΩ. Patch-clamp pipette was lowered into a slice preparation using a motorized manipulator (MX7600R, SD Instruments), current-clamp recordings were obtained using Multiclamp 700A amplifier (Molecular Devices), and the membrane potential was digitized using DigiData 1332A (Molecular Devices) at 10 kHz sampling rate. Positive and negative current steps were applied to characterize membrane properties of the neurons.

#### Extracellular Nerve Recordings From a Whole-Brain Preparation

Animals (*n* = 35) were anesthetized with MS-222, the brains were isolated in ice-cold saline oxygenated with 99% O_2_, and placed in a 100 mm Petri dish lined with Sylgard (Dow Corning) filled with oxygenated saline. After an hour of acclimation to room temperature (22°C), a brain was transferred to a recording chamber (Sylgard-lined 50 mm Petri dish, 20 mL total volume), and secured with fine minutien pins (Fine Science Tools). Except for 5-min period during which serotonin (5-HT, 60 μM) was applied to the brain, fresh oxygenated saline at room temperature was constantly superfused into the chamber at a rate of at least 150 mL/h.

Methods of recording the population activity of motor nucleus IX-X (n.IX-X) were as described previously (Rhodes et al., 2007). Briefly, fictive vocalizations were recorded from the most caudal root of cranial nerve IX-X via a suction electrode placed over the left and right nerves. This nerve root contains the axons of the laryngeal and glottal motoneurons (Simpson et al., 1986). The recorded compound action potentials (CAPs) were amplified (1,000×) with a differential A-C amplifier (model 1700; A-M Systems), high-pass filtered at 1 Hz, digitized at 10 kHz using Digidata 1440A (Molecular Devices), and recorded on a PC using PClamp (Molecular Devices). All the recordings were made at room temperature (22°C).

#### Data Analyses

For whole-cell patch-clamp recordings, resting membrane potential was taken as the average membrane potential over 1 min of recording in the absence of current injection, and input resistance was calculated from the steady-state membrane potential in response to small hyperpolarizing current pulses. Mann–Whitney *U* test was used to determine if the resting membrane potential and the input resistance differ between the labeled (infected by virus) and non-labeled (not-infected by virus) neurons. To examine if fictive vocalizations recorded from the VSV infected brains differ from those obtained from intact brains, we evaluated the synchronicity of the motor activity recorded from the right and left laryngeal nerves. We reasoned that if the infected side of the brainstem is dysfunctional, CAPs recorded from the infected side should lag behind the intact side (Yamaguchi et al., 2017). To this end, nerve recordings containing 10 consecutive fictive fast and slow trill CAPs from left and right nerves were used to calculate cross correlation coefficients. The time of the maximum cross-correlation coefficients (“the peak lag time”) of zero indicates synchronous activity of the two nerves. The peak lag time obtained from the infected brains and intact brains were compared using Mann–Whitney *U* test.

## Results

### Electroporation of Plasmids Drives Transgene Expression Mostly in Glial Cells, but Not in Neurons of Adult *X. laevis*

**FIGURE 2:**
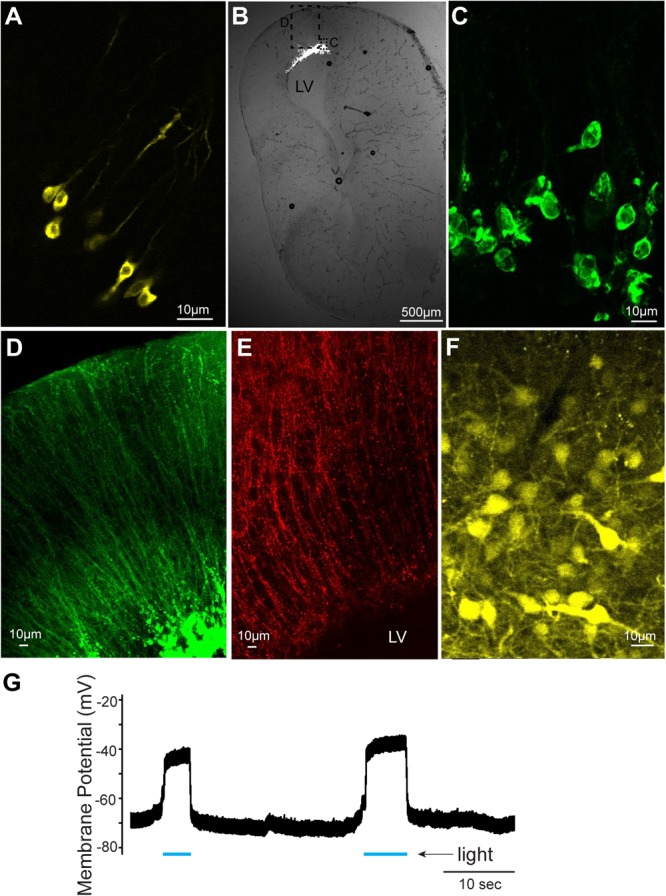
Cells labeled with fluorescent proteins after electroporation and Adeno-associated virus (AAV) injection. **(A)** Yellow fluorescent protein (YFP)-labeled tadpole neurons 1 day after plasmids were electroporated *in vivo*. **(B)** Transverse section of left telencephalon of adult *X. laevis* 5 days after plasmids were electroporated *in vivo*. LV indicates lateral ventricle. **(C)** Green fluorescent protein (GFP)-expressing cells (inset shown in **C**) that lack processes of neurons, presumed to be glial cells. **(D)** Labeled processes of glial-like cells (inset shown in **C**) that seem to form the ependymal lining of the ventricle. **(E)** Vimentin-positive radial glial cells in the adult telencephalon. **(F)** YFP-labeled telencephalic neurons of one of the very few brains that expressed reporter genes in response to AAV injection. **(G)** Membrane potential of a presumed glial cell electroporated with plasmid (pCS2FA.ChR2.YFP) positive for YFP and Channelrhodopsin 2 (ChR2). In response to blue light exposure (indicated in blue lines below the trace), the membrane potential depolarized, presumably because of the ChR2 expression, but the cell never spiked an action potential.

In this study, we first confirmed that plasmids can be electroporated into neurons of tadpoles (stage 47, *n* = 31, Figure 1A and Table 1); in all tadpoles, reporter gene expression in neurons was confirmed within 16 to 24 h of electroporation (100% success rate, Figure 2A) regardless of the types of plasmid electroporated (Tables 1, 2). Next, the same technique was applied to the brains of adult *X. laevis*. Taking advantage of the fact that the isolated brains of adult *X. laevis* survive for up to 7 days *in vitro* when stored at 4°C, we first electroporated plasmid into the adult brains *in vitro* (Figure 1B, *n* = 11). One to 3 days post electroporation, we detected reporter gene fluorescence in 90% of electroporated brains. However, unlike in tadpole brains, there were very few neurons that were labeled in adult electroporated brains. Instead, much of the fluorescence was observed along the lateral ventricle and subventricular zones with processes spanning the entire width of the telencephalon that resemble those of radial glial cells (data not shown).

To determine if this preferential electroporation of plasmids into glial cells is specific to electroporating conditions *in vitro*, we next electroporated plasmids in adult brains *in vivo* (Figure 1C, *n* = 8, Table 1). Five to six days post electroporation, four of eight animals did not show any reporter gene expression (50%). In the remaining four animals, we observed a pattern of reporter gene expression similar to those observed in the *in vitro* electroporated brains (Figures 2B–D). Morphologically, these labeled processes resemble radial glial cells, which serve as primary progenitor cells in the developing vertebrate CNS, but are known to persist in mature amphibians (D’Amico et al., 2013). Using two adult males, we verified that these processes are indeed those of radial glial cells by performing immunohistochemistry for vimentin, an intermediate filament protein that serves as a radial glial cell marker (Figure 2E). Furthermore, we carried out whole-cell patch-clamp recordings from subventricular zone cells transfected with pCS2FA.ChR2.YFP using brain slice preparations obtained from two animals. Current-clamp recordings from these cells showed that membrane potential depolarized in response to exposure to a blue, but not to a green light, verifying the expression of channelrhodopsin 2 in the membrane of the transfected cell (Figure 2G). However, these cells failed to fire action potentials in response to light nor to the depolarizing current injection, indicating that these cells are not neurons. Thus, the results show that when plasmids are electroporated into adult brain of *X. laevis*, transgene expression is preferentially seen in the glial cells, and not in neurons.

### Adeno-Associated Virus (AAV), Lentivirus (LV) Are Not Efficient Vectors for Adult *Xenopus* Nervous System

We next tested if AAV and LV can be used to deliver transgenes into adult *X. laevis* neurons. When AAV was injected into the telencephalon of adult *X. laevis*, the probability of infecting neurons was very low. Out of 49 adult *X. laevis* injected with AAV (Tables 1, 2), two animals (injected with AAV9.hSyn.hChR2) showed robust reporter gene expression in neurons 3 weeks post injection (Figure 2F). None of the remaining animals, including 13 animals injected with the same virus, showed expression in neurons after the same post-infection period (4% success rate). It was not clear what distinguished successful transduction from non-successful ones, but this low probability led us to conclude that AAV is not a reliable vector that can be used for the nervous system of adult *X. laevis*.

We next examined if LV can efficiently transduce neurons of adult *X. laevis*. All LV used was pseudotyped with vesicular stomatitis virus glycoprotein (VSV-G) which is reported to have a very broad tropism in the nervous system of vertebrate and invertebrate species (Mundell et al., 2015). When LV (Tables 1, 2) was injected into the telencephalon or optic tectum (*n* = 4) of adult *X. laevis*, however, none of the animals expressed reporter genes 6 weeks post infection (data not shown). Although the absence of reporter gene expression does not allow us to distinguish between inefficient infection and inefficient expression, we reasoned that LV pseudotyped with VSV-G should infect frog neurons, and thus gene expression following the infection is likely inefficient. To this end, we tested another type of LV, Equine infection anemia virus (EIAV), specifically engineered to drive robust transgene expression by using a positive feedback loop composed of a Tet promoter driving channelrhodopsin-2-YFP (Cetin and Callaway, 2014). Injection of this engineered EIAV (pseudotyped with VSV-G) into the forebrain of tadpoles (*n* = 9) and adult *X. laevis* (*n* = 9), however, did not result in reporter gene expression in neurons of any of the animals 12 days (tadpoles) or 12 weeks (adults) after injection (0% success rate, data not shown). Thus, we concluded that lentiviral vectors are ineffective for neurons of tadpole or adult *X. laevis*, despite the broad tropism endowed by VSV glycoproteins.

### Vesicular Stomatitis Virus Drove Robust Reporter Gene Expression in Neurons of Tadpole and Adult *Xenopus laevis*

**FIGURE 3:**
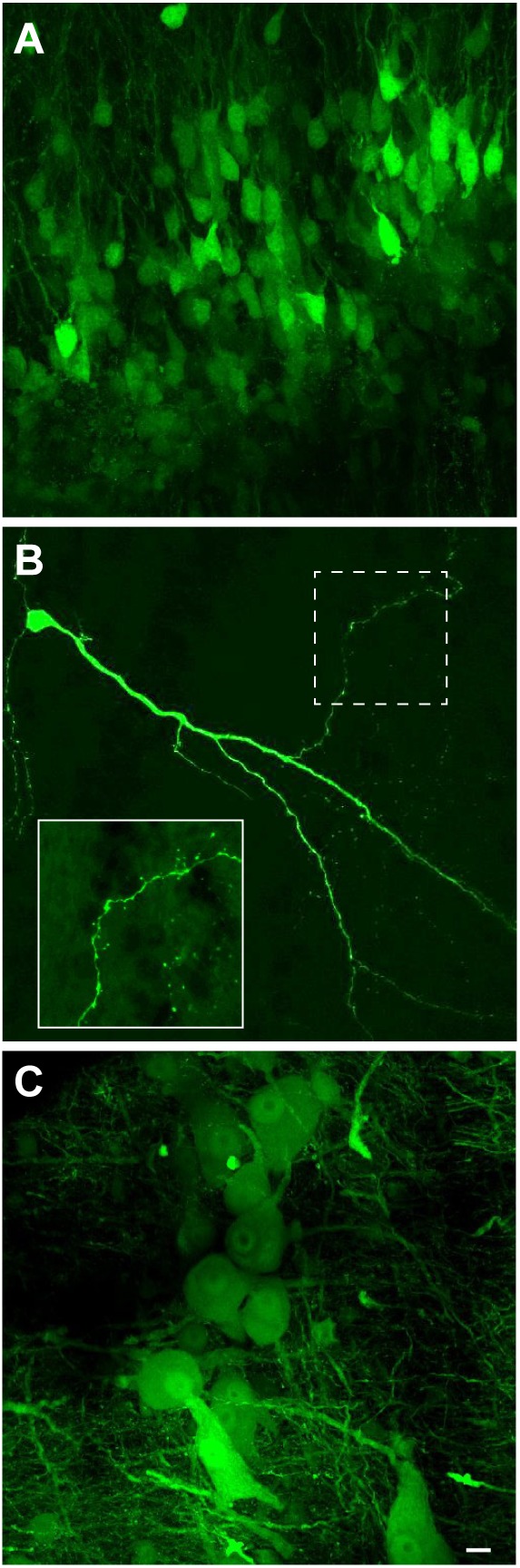
Neuroanatomical evidence that recombinant vesicular stomatitis virus encoding its own glycoprotein [rVSV(VSV-G)] transduce adult *X. laevis* neurons (i.e., transgene was moved from the virus to the neurons). **(A)** Adult *X. laevis* neurons in telencephalon expressing reporter gene (Venus 2) 48 h after injection of rVSV(VSV-G) plaque 21. Transduction efficiency was 86% (24/28 animals). **(B)** A lone infected neuron in optic tectum away from the injection site with a robust expression of reporter gene in dendrites, axons, dendritic spines and synaptic boutons observed 68 h after injection of rVSV(VSV-G) plaque 21. A dotted area is enlarged in the bottom left. **(C)** Neurons in laryngeal motor nucleus (n.IX-X) expressing Venus 2 48 h after injection of rVSV(VSV-G) plaque 21. The transduction efficiency in the brainstem with this strain of virus was 50% (9/18 animals). Scale bar is 10 μm, and applies for all three panels.

We next examined if VSV can transduce neurons of *X. laevis*. When rVSV encoding Venus 1 or 2 and its own glycoprotein [rVSV(VSV-G)] in the genome (Table 2) was injected into the olfactory bulb and optic tectum of tadpoles (*n* = 42) *in vivo*, robust reporter gene expression was observed as early as 6 h post injection in 93% of animals (39/42, data not shown). Similarly, when rVSV(VSV-G) was injected into the telencephalon, optic tectum, or olfactory bulb of adult frogs (*n* = 36) *in vivo* (Table 1), reporter gene expression was observed in 30 animals (83%) as early as 2 days post infection (Figure 3A), and stable expression of reporter gene was observed up to 10 days post infection. Consistent with previous observations made in mice neurons (van den Pol et al., 2009; Beier et al., 2013), transgene expression was detected not only in soma, but also in axons, dendrites, and dendritic spines; a lone infected neuron sporadically found away from the injection site had labeled dendrites, axons, dendritic spines and synaptic boutons (Figure 3B). The two plaque purified stocks of rVSV(VSV-G) used in this experiment, plaque purified isolate 14 and 21 (created by Beier et al., 2013, Table 1), infected olfactory bulb, telencephalon, and optic tectum of animals at a similar probability of 75% (*n* = 8) and 86% (*n* = 28) for plaques 14 and 21, respectively.

To test if neurons in brain regions rich in myelinated axons also can be transduced by rVSV(VSV-G) with an efficiency similar to those observed in the forebrain and midbrain of adults, we injected the virus into the laryngeal motor nucleus (n.IX-X) in the brainstem of adult male *X. laevis* (*n* = 24). Injection of rVSV(VSV-G) plaque 21 into the adult n.IX-X resulted in the reporter gene expression (Figure 3C) in 50% (9/18) of brains, a lower success rate than that observed in the region rich in cell bodies such as forebrain (86% success rate). Surprisingly, injection of rVSV(VSV-G) plaque 14 into n.IX-X resulted in no reporter gene expression in any of the brains (0/6), indicating that the two plaque purified stocks of rVSV(VSV-G) do not function equally in the brainstem, and that the transduction efficiency of rVSV(VSV-G) is region-specific within the brains of adult frogs.

We next explored whether variation in glycoproteins modifies the probability of transduction of *X. laevis* neurons by VSV. First, we used rVSV encoding eGFP with rabies glycoprotein (RABV-G) in the genome replacing VSV-G [rVSV(RABV-G)]. Injection of rVSV(RABV-G) into the olfactory bulb of tadpoles (*n* = 12) and telencephalon of adults (*n* = 7) resulted in reporter gene expression in 33% of tadpoles (4/12) 3 days post infection, and in 71% of adults (5/7) 1 to 3 days post infection (data not shown). Similarly, injection of rVSV(RABV-G) into the n.IX-X of adult male brains (*n* = 8) resulted in smaller proportion of animals with reporter gene expression (25%, 2/8). These results indicate that rVSV(RABV-G) can also infect neurons of *X. laevis*.

Second, we used glycoprotein gene deleted rVSV with inserted transgene encoding either tdTomato alone [rVSVΔG(VSV-G)tdTomato] or the combination of mCherry and Channelrhodopsin 2 fused to yellow fluorescent protein [rVSVΔG(VSV-G)ChR2YFP]. In these rVSVs, the VSV-G gene was removed from the viral genome, but was provided in trans from a G-expressing cell line during the virus stock preparation. Injection of both rVSVΔG(VSV-G) (Tables 1, 2) into the olfactory bulb, telencephalon, and optic tectum of the adult *X. laevis* (*n* = 10) resulted in reporter gene expression in 9 of 10 animals by 2 to 5 days post infection. When rVSVΔG(VSV-G) was injected into the n.IX-X of the adult male *X. laevis* (*n* = 3), robust reporter gene expression was observed in all three animals 2 to 10 days post infection. Taken together, all VSVs that we tested in this study can be used as robust tools to deliver genes into the neurons of adult *X. laevis*.

Interestingly, rVSVΔG(VSV-G) ChR2YFP, engineered by the Beier et al. (2013) (Table 2), encodes mCherry in the first position of the RNA genome (as with all the other rVSVs used in this study that has reporter gene insertion in the first position), and ChR2YFP in the fifth position. Adult *Xenopus* neurons transduced with the virus showed robust expression of mCherry, but the YFP signal was very faint if detected at all. Furthermore, when whole-cell patch-clamp recordings were obtained from the mCherry-labeled neurons using brain slice preparation of two animals, photostimulation of infected neurons did not result in depolarization of the membrane potential (data not shown), indicating that the expression of ChR2 gene inserted into the fifth genome position of VSV is insufficient.

### Neurons Transduced With VSV Are Physiologically Functional

**FIGURE 4:**
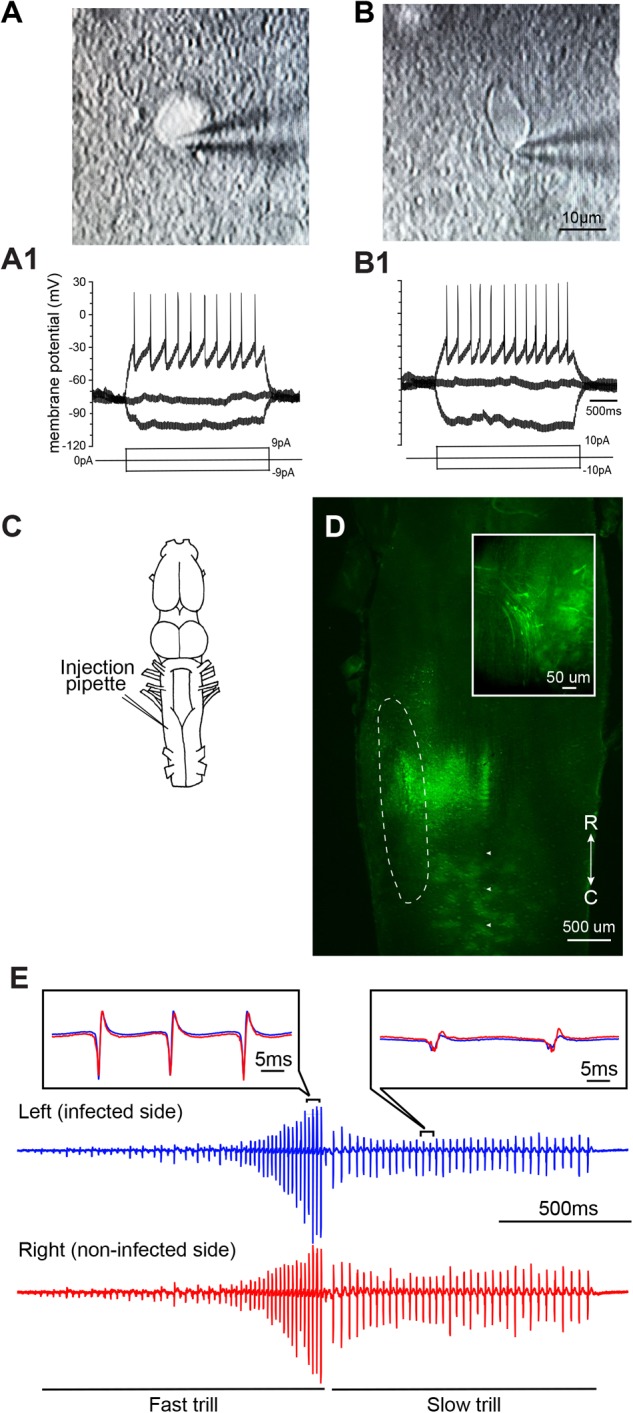
Neurons infected with vesicular stomatitis virus (VSV) are physiologically functional. **(A)** Differential interference contrast (DIC) image of an adult *X. laevis* telencephalic neuron expressing reporter gene (enhanced green fluorescent protein, eGFP) in a whole-cell patch-clamp configuration. The brain was injected with recombinant VSV encoding rabies glycoprotein [rVSV(RABV-G)] 2 days prior to the recordings was obtained. **(A1)** Membrane potential in response to current injections into the neuron shown in **(A)**. **(B)** A DIC image of a non-labeled telencephalic neuron near the neuron shown in **(A)** on the same slice preparation. **(B1)** Membrane potential of neuron B in response to current injection. **(C)** Schematic diagram illustrating the unilateral injection of recombinant vesicular stomatitis virus encoding its own glycoprotein [rVSV(VSV-G)] into the laryngeal motor nucleus, n.IX-X. **(D)** Horizontal section of the brainstem showing the labeled neurons in the n.IX-X (encircled in dotted white line) 2 days after rVSV(VSV-G) was injected. Reporter gene expression was observed in the somata and axons (inset) of vocal motoneurons. White arrowheads indicate the midline. **(E)** A fictive advertisement call elicited from the isolated infected brain shown in **(D)** in response to the application of serotonin (5-HT). The advertisement call is made of fast and slow trills (labeled below the traces). Extracellular recordings obtained from the left (blue) and right (red) laryngeal nerves are shown. Inset shows enlarged sections of the left and right extracellular recording traces superimposed to demonstrate the temporal synchrony.

To determine if neurons of adult *X. laevis* infected with VSV show pathological signs within the first days of transduction, we compared intrinsic properties of infected telencephalic neurons from two adult animals, one injected with rVSV(RABV-G) and the other with rVSVΔG(VSV-G). Two [rVSV(RABV-G) injected animal] and 7 days [rVSVΔG(VSV-G) injected animal] post infection, whole-cell patch-clamp recordings were obtained from labeled neurons (Figures 4A,A1) and their neighboring non-labeled neurons (Figures 4B,B1) using a brain slice preparation. Labeled neurons and non-labeled neurons had similar resting membrane potential (-41.30 + 1.98, -49.67 + 1.93 mV, mean ± SE. for labeled (*n* = 4) and non-labeled neuron (*n* = 3); Mann–Whitney *U* test, *Z* = -1.768, *p* = 0.077), and input resistance (1.42 + 0.74, 1.61 + 0.43 GΩ for labeled and non-labeled neurons; Mann–Whitney *U* test, *Z* = -0.722, *p* = 0.471). Current-evoked spike trains generated by these two groups were also similar (e.g., Figures 4A1,B1). Therefore, neurons transduced with VSV appear physiologically normal within the first 2 to 7 days of infection.

We next tested if VSV infection disrupts neural circuit function. The n.IX-X of the brainstem of adult *X. laevis* contains laryngeal motoneurons and interneurons that mediate vocal production (Brahic and Kelley, 2003). Previously, we have shown that application of serotonin (5-HT) to the isolated adult male brains elicits fictive advertisement calls (Rhodes et al., 2007). To test if the infection of vocal neurons in the n.IX-X by rVSV disrupts the function of the vocal neuronal circuitry, we used the brains of adult male *X. laevis* that showed positive reporter gene expression in the n.IX-X, as described above. These animals were injected unilaterally into the n.IX-X (Figure 4C) with one of the three VSVs (rVSV(VSV-G) plaque 21, *n* = 5; rVSV(RABV-G), *n* = 2; and rVSVΔG(VSV-G), *n* = 3). Two to 9 days post infection (Table 1), the brains of infected males were isolated, and 5-HT was applied *in vitro* while extracellular recordings were obtained from the laryngeal nerves bilaterally. In all of these brains, laryngeal motoneurons and interneurons in n.IX-X expressed reporter genes (Figure 4D), and labeled motor axons were also observed (Figure 4D inset). Fictive advertisement calls were elicited from the brains of 5 out of 10 animals [three with rVSV(VSV-G) plaque 21, one with rVSV(RABV-G), one with rVSVΔG(VSV-G), injection, Figure 4E]. Specifically, fictive advertisement calls were elicited from all three rVSV(VSV-G) injected brains 2 days post infection, and from the remaining two brains infected with rVSV(RABV-G) and rVSVΔG(VSV-G) 9 days post infection. In male *X. laevis* brains, two separate vocal central pattern generators (CPGs) are contained in the left and right half of the brainstem; when activated, the right CPG can drive the left motoneurons with a slight delay, even when the left CPG is silent (Yamaguchi et al., 2017). To determine if the infected side of the brainstem is dysfunctional but driven by the CPG in the non-infected side, we evaluated the synchronicity of CAPs recorded from the two laryngeal nerves by obtaining the peak time lag based on cross correlation coefficients (see “Materials and Methods”). The results showed that CAPs recorded from the VSV infected brains were largely synchronous (Figure 4E insets), and had a similar peak time lag to those obtained from uninfected brains (fast trill peak lag time; *Z* = -1.461, *p* = 0.144, slow trill peak lag time; *Z* = -0.730, *p* = 0.465, fast trill maximum cross correlation coefficient; *Z* = -0.548, *p* = 0.584, slow trill maximum cross correlation coefficient; *Z* = -0.548, *p* = 0.584, Mann–Whitney *U* test). Thus, VSV infected brains generated synchronous CAPs from the two sides of the brain, indicating that infection did not impair the function of the vocal CPG on the infected side of the brainstem. Given that fictive advertisement calls are elicited from ∼70% of the brains isolated from intact male *X. laevis* in our laboratory, a 50% success rate for 5-HT-induced vocalization is comparable to those obtained from the intact animals. Therefore, we conclude that infection with VSVs does not prevent the vocal neural circuits from functioning normally to generate fictive advertisement calls for up to 9 days after infection.

### The Infection Site of the Host Neurons by the VSVs Include Soma and Axon Terminals

**FIGURE 5:**
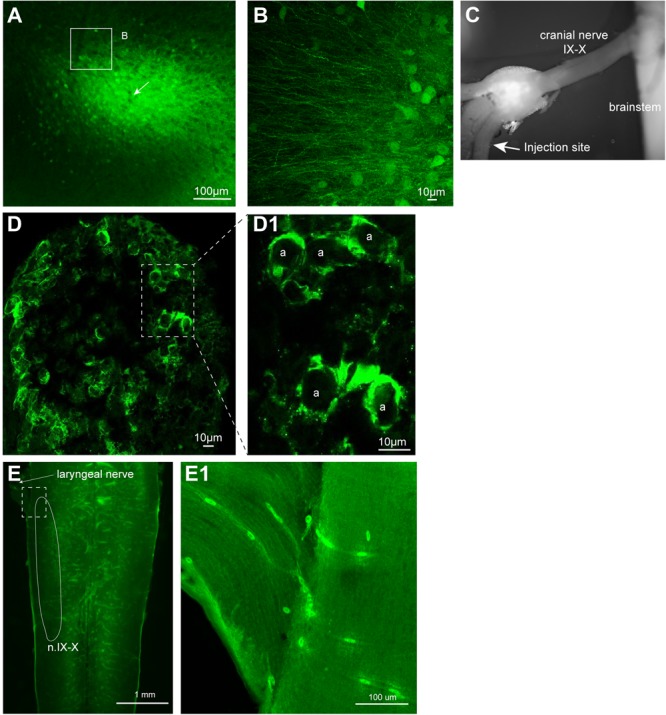
Neurons transduced by vesicular stomatitis virus (VSV) around the injection site. **(A)** Neurons with reporter gene expression near the injection site of recombinant vesicular stomatitis virus encoding its own glycoprotein [rVSV(VSV-G)] in the telencephalon 2 days post infection. There was a robust expression of reporter genes about 400 μm surrounding the injection site. A white arrow indicates the injection site. **(B)** In addition to labeled somata, there are dense processes that are labeled around the injection site. **(C)** The nerve injection site shown in the isolated brain. **(D)** Cross section of the laryngeal nerve 24 days post injection. **(D1)** Enlarged section of perforated rectangle shown in **(E)**. Note that the labeling is concentrated on the periphery of the axons (a, in inset on the right). **(E)** The horizontal section of the brainstem of a male *X. laevis* with rVSV(VSV-G) injected into the cranial nerve IX-X 24 days prior. The white line encircle the n.IX-X. There was no labeled neurons nor axons found in the brainstem or the nerve. **(E1)** Enlarge section of a white perforated rectangle shown in **(E)**. Labeled cells seen are autofluorescent red blood cells in the blood vessels (amphibian red blood cells are nucleated).

Once we established the effectiveness of VSVs in infecting neurons of adult *X. laevis*, we next explored the location of viral uptake by the host neurons. Consistent with the previous report on mice, we found many somata of neurons near the injection site expressing reporter genes. For example, when 500 nL of rVSV(VSV-G) (*n* = 14) or rVSV(RABV-G) (*n* = 4) were injected into forebrain and midbrain of adult *X. laevis*, labeled cell bodies were found mostly within 400 μm of the injection site 2 days post infection (Figure 5A). In addition to labeled somata, there were a large number of labeled processes near the injection site (Figure 5B). These processes may be dendrites and axons of the directly infected neurons. Alternatively, VSV may infect axons at the injection site. A previous study showed that VSV does not infect axons of passage in mammals; injection of replication-deficient rVSVΔG into corpus callosum of mice that contain axons that connect right and left cortex did not result in labeled cortical neurons (van den Pol et al., 2009).

To examine if VSV also fails to infect axons of passage in frog nervous system, rVSV(VSV-G) plaque 21 was injected directly into the nerve IX-X, a cranial nerve that carry axons of laryngeal motoneurons (Table 1). If rVSV(VSV-G) infects axons, we predict to see reporter gene expression in the laryngeal motoneuron axons and somata. In one group of animals, the injection was made 5 mm afferent from the laryngeal muscles (*n* = 3), and in another group of animals, the injection was made 3 mm efferent from the brainstem (*n* = 4, Figure 5C). Cross-section of the laryngeal nerve 2 to 35 days post injection (Table 1) showed some reporter gene expression (Figure 5D). However, labeling appeared to be concentrated in the periphery of the axons (Figure 5D1), suggesting the virus may have infected myelinating oligodendrocytes, but not the axons. Consistent with this observation, no labeled laryngeal motoneuron somata were found in the brainstems (Figure 5E) or the laryngeal nerve (Figure 5E1) of any of the animals. These results indicate that rVSV(VSV-G) does not efficiently infect axons of passage.

**FIGURE 6:**
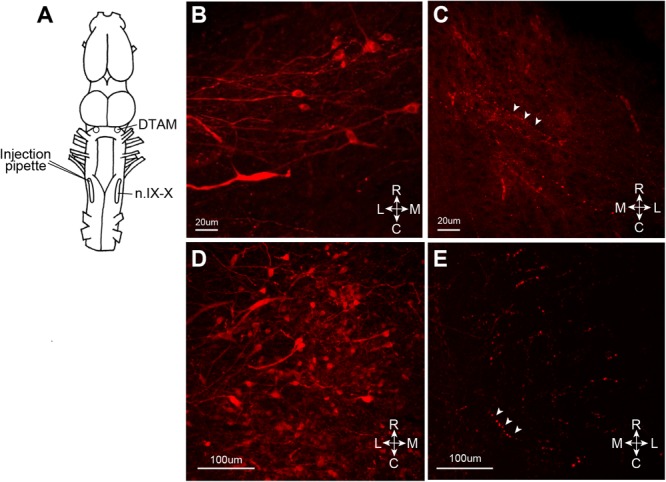
Retrograde infection of recombinant vesicular stomatitis virus (VSV) missing the glycoprotein (G) gene from its genome, but pseudotyped with the wild-type VSV-G [rVSVΔG(VSV-G)]. VSV infect the neurons via axon terminal. **(A)** A cartoon showing the dorsal view of the *X. laevis* brain with the unilateral injection of rVSVΔG(VSV-G) into the left anterior laryngeal motor nucleus (n.IX-X). **(B)** Nine days after injection, labeled somata with lateral axonal projections were found in ipsilateral dorsal tegmental area of medulla (DTAM). **(C)** In the contralateral DTAM, there were no labeled neuron somata but some labeled processes (white arrow heads). **(D)** Left n.IX-X injected with rVSVΔG(VSV-G) shows a large number of labeled neurons. **(E)** The contralateral n.IX-X show no labeled neurons, but labeled axons are seen (white arrow heads).

We next asked whether the VSV can infect neurons via their axon terminals. To explore the possibility of retrograde infection by VSV, we injected replication deficient rVSVΔG(VSV) into the laryngeal motor nucleus (n.IX-X) of adult male *X. laevis* (*n* = 3, Table 1). Neurons in n.IX-X of adult male *X. laevis* send and receive reciprocal synaptic connections with the dorsal tegmental area of medulla (DTAM, a homolog of parabrachial nucleus in mammals) located 2.5 mm rostral to the nucleus (Figure 6A). Thus, we reasoned that transgene expression in DTAM neurons would indicate retrograde infection of these neurons via their axon terminals in n. IX-X. We found a large number of labeled somata (Figure 6D) within ∼750 μm of the injection site in n.IX-X in all three animals. In addition, in two out of three animals, labeled cell bodies of neurons were found in DTAM of the ipsilateral side (Figure 6B). The labeled DTAM neurons had axons that projected laterally from the cell bodies (Figure 6B), a characteristic of the n.IX-X projecting DTAM neurons (Zornik and Kelley, 2007). There were no other population of labeled neurons between the injection site and ipsilateral DTAM. Contralateral n.IX-X that is ∼2 mm away from the injection site and contralateral DTAM that is ∼4 mm away from the injection site showed some labeled processes (presumably of the projection neurons in the injected n.IX-X, Figures 6C,E, white arrow heads) but no labeled somata (Figures 6C,E). Taken together, the result suggests that the rVSVΔG(VSV) infect the axon terminals of adult *X. laevis* neurons.

### Transsynaptic Infection by VSV Was Not Observed in *X. laevis*

**FIGURE 7:**
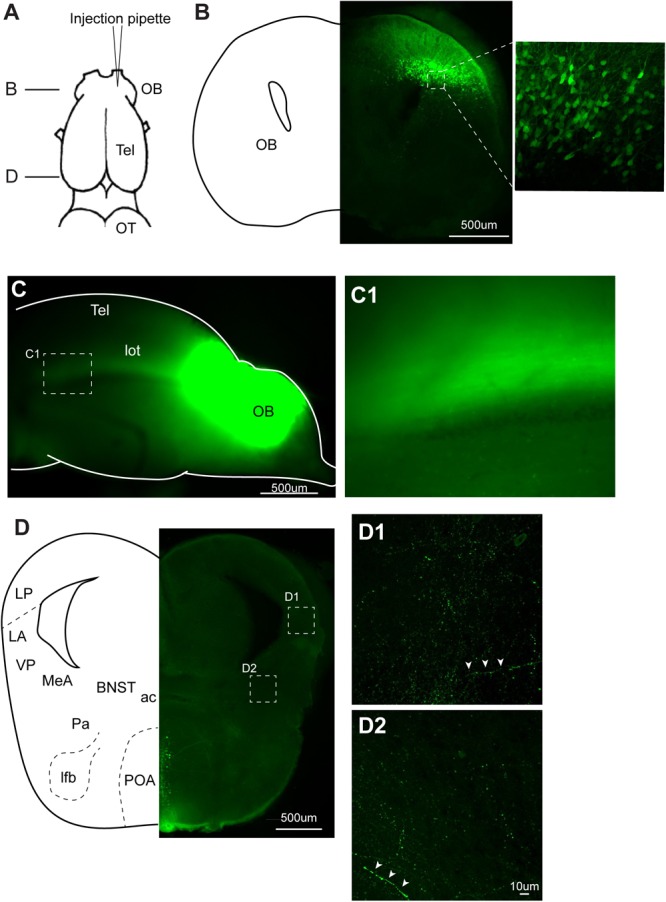
Recombinant vesicular stomatitis virus encoding VSV glycoprotein [rVSV(VSV-G)] does not spread across axons in anterograde direction in the central nervous system of *Xenopus laevis*. **(A)** A dorsal view of the *X. laevis* forebrain illustrating the injection site of the virus into olfactory bulb (OB). Tel, telencephalon; OT, optic tectum. **(B)** A coronal section of the olfactory bulb at the rostral-caudal level shown in **(A)**, showing a large number of labeled neurons in the injected side 10 days post injection. The inset shows an enlarged section showing labeled neurons. **(C)** A lateral view of the left olfactory bulb and left telencephalon in which VSV was injected 10 days prior. Lateral olfactory tract (lot) is visible from the surface of the telencephalon. **(C1)** An enlarged view of the lateral olfactory tract shown in **(C)**. **(D)** A coronal section of the caudal telencephalon at the rostral-caudal level shown in A. No labeled neurons were found either in lateral amygdala **(D1)** or in medial amygdala **(D2)**. White arrow heads show labeled processes. Labeled neurons seen in preoptic area (POA) are considered to have resulted from the intracranial leak of the injected virus from the injection site in the olfactory bulb. ac, anterior commissure; BST, bed nucleus of the stria terminalis; LA, lateral amygdala; lfb, lateral forebrain bundle; LP, lateral pallium; MeA, medial amygdala; Pa, pallidum; POA, preoptic area; VP, ventral pallidum.

To test if VSV spread across synapses of adult *Xenopus* neurons, as previously reported in mice, we first injected rVSV(VSV-G) unilaterally into olfactory bulb of adult *Xenopus laevis* (Figure 7A, *n* = 9). Neurons in the main and accessory olfactory bulb project to lateral/medial amygdala in *X. laevis* (Moreno and Gonzalez, 2006), but there is no reciprocal connection from the amygdala to the olfactory bulb (Moreno and Gonzalez, 2003). Given the distance between the olfactory bulb and the amygdala (4.0 mm), we reasoned that labeled neurons in amygdala after olfactory bulb VSV injection would suggest spread of rVSV(VSV-G) across synapses in the anterograde direction. When examined 10 days post-injection, neurons in the olfactory bulbs were brightly labeled in five of the nine animals (Figure 7B), and labeled lateral olfactory tract was visible from the lateral surface of the telencephalon (Figures 7C,C1). However, we found no labeled somata in the medial or lateral amygdala region of these brains, although labeled processes and terminal fields of the presumed projection neurons from the olfactory bulb were apparent (Figures 7D,D1,D2 white arrow heads). The result is consistent with the idea that rVSV(VSV-G) does not spread across synapses in the anterograde direction in the CNS of adult *X. laevis*.

**FIGURE 8:**
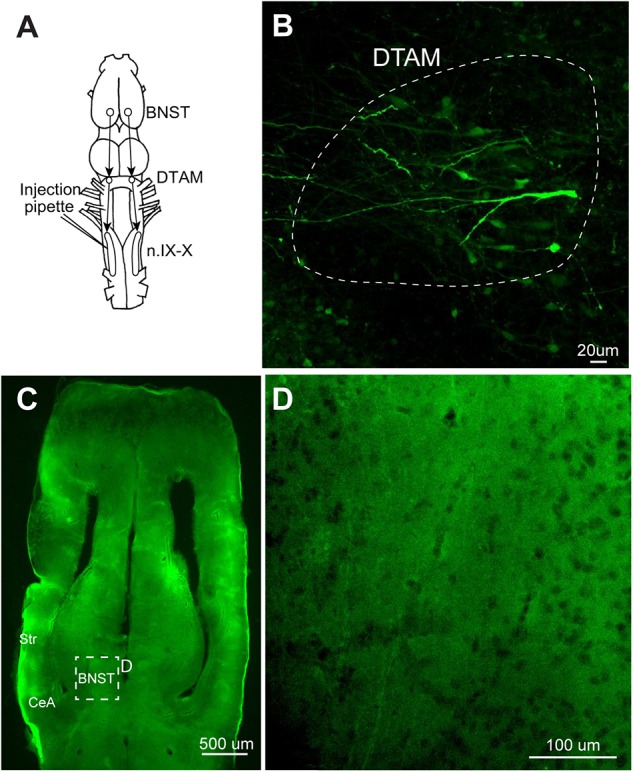
A test for the transsynaptic spread of recombinant vesicular stomatitis virus encoding rabies glycoprotein [rVSV(RABV-G)]. **(A)** A dorsal view of the *X. laevis* forebrain illustrating the connection among laryngeal motor nucleus (n.IX-X), dorsal tegmental area of medulla (DTAM) and the bed nucleus of the stria terminalis (BNST); n.IX-X receives projections from ipsilateral DTAM, which in turn, receives input from the ipsilateral bed nucleus of the BNST. **(B)** A horizontal section of the anterior brainstem showing labeled neurons in ipsilateral DTAM 10 days post infection. White dotted line encircle DTAM. **(C)** Horizontal section of the caudal telencephalon showing the location of BNST. **(D)** An enlarged section in C showing the absence of labeling in the ipsilateral BNST 10 days post injection.

We next examined retrograde transsynaptic transfer of rVSV(RABV-G). To test this possibility, we first injected rVSV(RABV-G) into the laryngeal muscles (*n* = 10) and gastrocnemius muscles (*n* = 4) of adult *X. laevis* (Table 1). Infection of the muscle fibers followed by the spread across synapse to presynaptic motoneurons should result in labeled motoneurons in both cases. Seven to 28 days post injection (Table 1), we found no labeled motor axons or motoneurons somata in any of the animals examined (data not shown). Furthermore, none of the muscle fibers injected except for a single fiber (gastrocnemius) in one animal were labeled. To further address this question of retrograde transsynaptic spread, we injected rVSV(RABV-G) into the n.IX-X of adult male *X. laevis* (*n* = 8, Table 1). In adult male *X. laevis* brains, neurons in the bed nucleus of the stria terminalis (BNST) project to DTAM (Hall et al., 2013), which in turn project to n.IX-X (Figure 8A). In two out of the eight animals, labeled neurons were found both in the injection site of n.IX-X (data not shown) and in the DTAM ipsilateral to the injection site (Figure 8B) in 8 (*n* = 1) or 9 (*n* = 1) days post injection. However, there were no labeled neurons in the BNST in either animal (Figures 8C,D). These result suggest that rVSV(RABV-G) does not spread across synapses retrogradely in the adult *X. laevis* CNS.

## Discussion

We tested electroporation and viral vectors to introduce transgenes into adult neurons of adult *X. laevis*. We found that VSV is a quick, efficient, and robust viral vector that can be used reliably to transduce neurons of adult *X. laevis* while other methods (electroporation, AAV, and LV) were either unreliable or entirely ineffective in introducing transgenes in adult *X. laevis* neurons.

The failure of electroporation to drive transgene expression in adult *X. laevis* was surprising given its success in tadpoles and in a variety of other vertebrate systems. One alternate explanation for these results may be that the activity of CAG and the CMV promoters used to drive reporter gene expression are ineffective in adult *X. laevis* neurons. However, in a transgenic *X. laevis* created by Sakamaki et al. (2005), both CAG and CMV promoters were shown to drive the reporter gene expression in the neurons of both tadpoles and post-metamorphic juvenile *X. laevis* frogs, suggesting that these promoters remain active throughout the development. Furthermore, when transduced or transfected acutely, both promoters drive gene expression not only in the neurons of adult mammals, but also in adults of other vertebrate species including zebrafish (Zou et al., 2014) (but see, Rothenaigner et al., 2011), axolotls and zebra finches (Heston and White, 2017). Thus, we conclude instead that our results reflect difference in the transfection efficiency of electroporation between adult and tadpole neurons.

The electrical field and transmembrane voltage experienced by adult neurons may be significantly different from those experienced by tadpole neurons. Although the larger cells in adults should be easier to electroporate than the smaller cells because transmembrane voltage is a function of the cell radius (Canatella et al., 2004; Luft and Ketteler, 2015), our results were the opposite. Changes in the cell density and extracellular matrix associated with the maturation of the nervous system may modify the effectiveness of generating the transmembrane voltage necessary for successful electroporation in adult *X. laevis*. In addition, adult neurons may differ in their cell architecture and biochemistry from those of tadpoles (Canatella et al., 2004). Although we were not able to electroporate adult neurons in this study, we showed successful electroporation of glial cells in adult *X. laevis*, which may be useful for other fields of study, such as adult neurogenesis.

Adeno-associated virus and LV are the most commonly used viral vectors for introducing transgenes into the intact nervous system. Unfortunately, we found that these tools are largely ineffective in *Xenopus laevis*. Unlike VSV, both AAV and LV require promoter-mediated transcription for their reporter gene expression. While we cannot rule out the possibility that the promoters used in these viruses (CAG, CMV, and human synapsin, hSyn) had low activity in adult *X. laevis* neurons, for reasons discussed above, we believe that a more likely explanation is that in majority of the AAV injected animals, AAV failed to infect neurons. Successful infection of a host cell by AAV requires entry into the cell followed by intracellular trafficking of AAV into the nucleus (Pillay et al., 2017). Notably, a human homolog of the AAV receptor gene (AAVR, also known as KIAA0319L) (Pillay et al., 2017) identified in the *Xenopus laevis* database only has 63% identity to the human sequence.

The reasons for ineffective transduction by the VSV-G pseudotyped lentivirus are less clear. Given the wide host range of VSV-G, it is likely that VSV-G coating the LV bound to the *X. laevis* neurons. However, even Tet-Loop amplified EIAV failed to transduce *X. laevis* neurons, suggesting that the problem likely has to do with post-infection intracellular processes prior to gene expression in *X. laevis* neurons. Temperature may play an important role in LV transduction; injection of LV pseudotyped with VSV-G in to the brains of adult zebrafish followed by housing at 37°C for 24 h resulted in successful transduction of neurons (Rothenaigner et al., 2011).

Vesicular stomatitis virus transduced neurons of tadpoles and adult *X. laevis* with high probability, and led to robust transgene expression. A variety of recombinant VSVs used in this study were effective. Our results suggest that the transduction rate of neurons by VSVs (measured by the proportion of animals with a positive reporter gene expression) may be higher in the fore- and midbrain than in hindbrain of adult *X. laevis*, although quantitative analyses with a larger sample size is necessary in the future to clarify this point.

In cultured embryonic mice neurons, VSV reporter gene expression was detected in as little as an hour after inoculation (van den Pol et al., 2009). Although we did not systematically quantify reporter gene expression over time in infected frog neurons, we observed neurons with strong expression in all cellular compartments including presumed synaptic boutons and dendritic spines within 3-days post infection (Figure 3B). In some brightly labeled neurons, Venus and tdTomato reporter proteins that should remain in cytosol seem to fill the entire soma including cell nuclei, as has been observed in VSV-infected mice neurons in other studies (van den Pol et al., 2009; Beier et al., 2011, 2013; Mundell et al., 2015). Although convenient, excessive levels of reporter gene expression likely disrupt the health of host neurons. Even without the excessive reporter gene products produced by recombinant VSV, wild-type VSV is known to be toxic to the host cells by blocking regular cellular processes (van den Pol et al., 2009; Wollmann et al., 2010). Thus, neurons infected with recombinant VSVs likely experience two types of health defects; one resulting from VSV interfering with regular cellular processes, and the second resulting from excessive production of the reporter proteins.

Nonetheless, infected frog neurons showed normal electrophysiological properties for days after transgene expression was detected. Two [rVSV(RABV-G)] and 7 days [rVSV1G (VSV-G)] post infection, intrinsic and firing properties of the infected neurons labeled with reporter gene were not significantly different from uninfected neurons. Moreover, fictive vocalizations were recorded from the brains infected with rVSVΔG (VSV-G) up to 9 days post infection, suggesting that neural circuits containing VSV-infected neurons remain intact and functional long after infection. Previous studies in mice have reported that electrophysiological properties of mouse neurons inoculated with either rVSVΔG or rVSV(RABV-G) remain normal up to 16 h post infection, but that these deteriorate after day 2 (van den Pol et al., 2009; Beier et al., 2013). This striking discrepancy between the results obtained in mice and *X. laevis* may be due to the difference in the virulence of the virus on host species; VSV may be more virulent to mammalian neurons than to amphibian neurons, potentially because of the lower body temperature in the latter. The longer window of time between the time of infection and the start of the deterioration of the cell in amphibians provides us with a greater opportunity to apply this genetic tool to physiological experiments.

The pattern of labeled neurons around an injection site suggests that the uptake of VSV take place most commonly at or near soma of adult *X. laevis* neurons. Consistent with a previous finding in mice (van den Pol et al., 2009; Beier et al., 2013), VSV was not taken up by the axon of passage in the injection site, but did appear able to infect neurons via their axon terminals. When replication deficient rVSVΔG(VSV) was injected into n.IX-X unilaterally, labeled cell bodies were observed in ipsilateral DTAM that is located 2.5 mm rostral from the injection site, that is known to contain projection neurons to n.IX-X. Since it is highly unlikely that the labeled DTAM neurons were infected by diffusion of replication deficient virus from the injection site, we conclude that the rVSVΔG(VSV) retrograde infect neurons of adult *X. laevis*, allowing the labeling of projection neurons in addition to local neurons. Viral infection via axon terminals (retrograde infection) is a part of the life cycle of some viruses including rabies (Ugolini, 2011) and polio virus (Ren and Racaniello, 1992), and has been reported in other viruses (Rothermel et al., 2013; Tervo et al., 2016). The ability of VSV to target both the neuronal cell bodies at the site of exposure and the projection neurons that sends axons to the injection site for transgene delivery is a powerful feature that will allow us to monitor and manipulate the activity of neurons to understand the function of the neural circuits, as has been done using AAV and LV specifically engineered to have the capability of retrograde infection (Tervo et al., 2016; Sheikh et al., 2018).

Previous studies reported that VSVs can spread across synapses, and the direction of the spread was determined by the identity of the glycoprotein; rVSV(RABV-G) showed retrograde transsynaptic spread whereas rVSV(VSV-G) exhibited anterograde transsynaptic spread (Beier et al., 2011, 2013, 2016; Mundell et al., 2015). However, we did not see any evidence of transsynaptic spread of VSV in frog neurons in either direction. The properties of VSV spread across synapses may differ between the host species; the efficiency of the spread may be significantly lower in amphibians. We conclude that rVSV(VSV-G) is not a tool that can be used to trace neural connectivity of adult *X. laevis*.

## Conclusion

We identified VSVs as a quick and efficient viral vector that results in robust gene expression in adult CNS of amphibian, *Xenopus laevis*. Remarkably, the infected neurons expressing exogenous gene remain functional for days in *X. laevis* CNS, and thus, it is feasible to apply genetic tools to physiological studies. The ability of VSVs to be taken up by the axon terminals of projection neurons allows monitoring and manipulation of a remote population of neurons that will facilitate the analyses of neural circuits. With a broad host range, VSV can be used to drive exogenous gene expression in a variety of species, and thus prove to be a powerful tool to analyze the nervous system of non-genetic model organisms.

## Author Contributions

AY designed the study, collected and analyzed data, and wrote the manuscript. DW collected and analyzed the data. PV collected the data. DW and PV edited the manuscript.

## Conflict of Interest Statement

The authors declare that the research was conducted in the absence of any commercial or financial relationships that could be construed as a potential conflict of interest.
